# Transcriptional Downregulation of miR-4306 serves as a New Therapeutic Target for Triple Negative Breast Cancer: Erratum

**DOI:** 10.7150/thno.82636

**Published:** 2023-02-09

**Authors:** Zitong Zhao, Lin Li, Peina Du, Liying Ma, Weimin Zhang, Leilei Zheng, Bo Lan, Bailin Zhang, Fei Ma, Bo Xu, Qimin Zhan, Yongmei Song

**Affiliations:** 1 State Key Laboratory of Molecular Oncology, National Cancer Center/National Clinical Research Center for Cancer/Cancer Hospital, Chinese Academy of Medical Sciences and Peking Union Medical College, Beijing 100021, China; 2 Breast Cancer Center and the Key Laboratory of Breast Cancer Prevention and Therapy, Tianjin Medical University Cancer Institute and Hospital, National Clinical Research Center for Cancer, Key Laboratory of Cancer Prevention and Therapy, Tianjin 300060, China; 3 Department of Medical Oncology, National Cancer Center/National Clinical Research Center for Cancer/Cancer Hospital, Chinese Academy of Medical Sciences and Peking Union Medical College, Beijing 100021, China; 4 BGI-Shenzhen, Shenzhen, Guangdong 518083, China; 5 Department of Breast Surgery, National Cancer Center/National Clinical Research Center for Cancer/Cancer Hospital, Chinese Academy of Medical Sciences and Peking Union Medical College, Beijing 100021, China; 6 Laboratory of Molecular Oncology, Peking University Cancer Hospital, Beijing 100142, China

We recently found that some pictures were mistakenly chosen and placed in Figure 5B, Supplementary Figure 1A and Supplementary Figure 4. The correct pictures are provided below. The revised figures do not alter the conclusions of this article. We apologized deeply for our careless and any inconvenience caused.

## Figures and Tables

**Figure 1:**
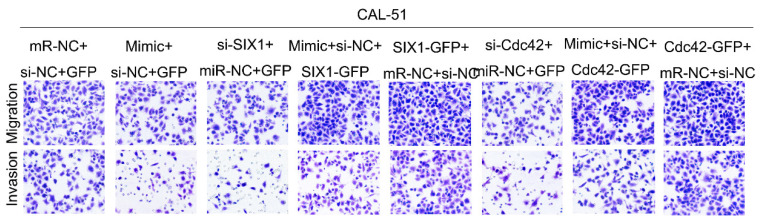
Correct image for original Figure 5(B) Transwell results for CAL-51 cells after different treatments.

**Figure 2:**
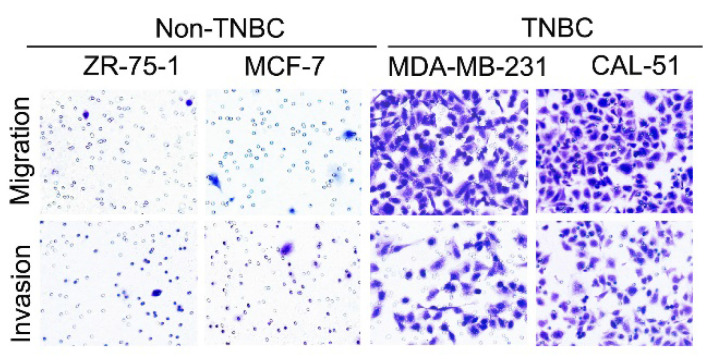
Correct image for original Supplementary Figure 1A Transwell results for breast cancer cells.

**Figure 3:**
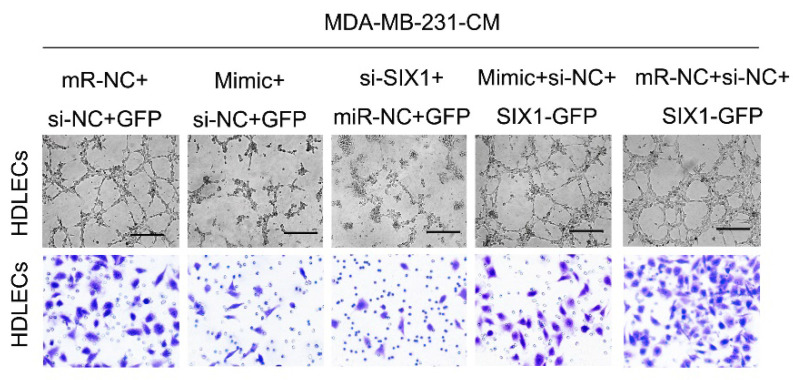
Correct image for original Supplementary Figure 4A Matrigel tube formation assay with different treatments.

**Figure 4:**
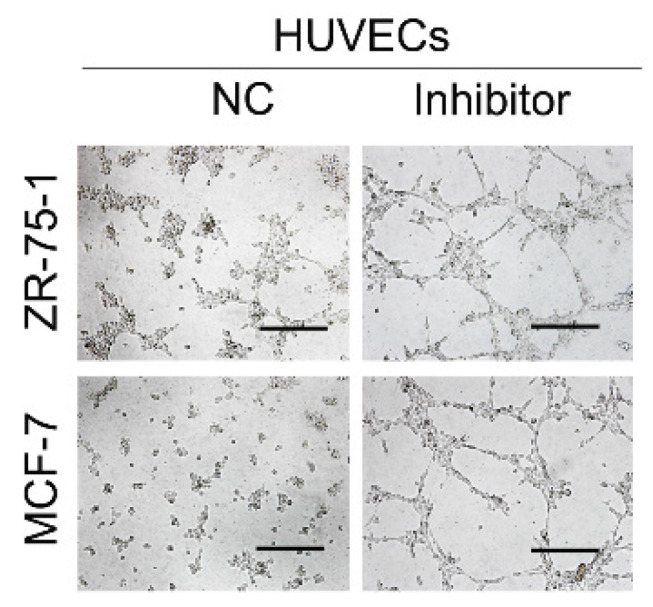
Correct image for original Supplementary Figure 4E Matrigel tube formation assay with different treatments.

